# Rate of Intensive Care Unit admission and outcomes among patients with coronavirus: A systematic review and Meta-analysis

**DOI:** 10.1371/journal.pone.0235653

**Published:** 2020-07-10

**Authors:** Semagn Mekonnen Abate, Siraj Ahmed Ali, Bahiru Mantfardo, Bivash Basu

**Affiliations:** 1 Department of Anesthesiology, College of Health Sciences and Medicine, Dilla University, Dilla, Ethiopia; 2 Department of Internal Medicine, College of Health Sciences and Medicine, Dilla University, Dilla, Ethiopia; 3 Department of Anesthesiology, College of Medicine, University of Calcutta, Calcutta, India; Azienda Ospedaliero Universitaria Careggi, ITALY

## Abstract

**Background:**

The rate of ICU admission among patients with coronavirus varied from 3% to 100% and the mortality was as high as 86% of admitted patients. The objective of the systematic review was to investigate the rate of ICU admission, mortality, morbidity, and complications among patients with coronavirus.

**Methods:**

A comprehensive strategy was conducted in PubMed/Medline; Science direct and LILACS from December 2002 to May 2020 without language restriction. The Heterogeneity among the included studies was checked with forest plot, χ2 test, I2 test, and the p-values. All observational studies reporting rate of ICU admission, the prevalence of mortality and its determinants among ICU admitted patients with coronavirus were included and the rest were excluded

**Result:**

A total of 646 articles were identified from different databases and 50 articles were selected for evaluation. Thirty-seven Articles with 24983 participants were included. The rate of ICU admission was 32% (95% CI: 26 to 38, 37 studies and 32, 741 participants). The Meta-Analysis revealed that the pooled prevalence of mortality in patients with coronavirus disease in ICU was 39% (95% CI: 34 to 43, 37 studies and 24, 983 participants).

**Conclusion:**

The Meta-Analysis revealed that approximately one-third of patients admitted to ICU with severe Coronavirus disease and more than thirty percent of patients admitted to ICU with a severe form of COVID-19 for better care died which warns the health care stakeholders to give attention to intensive care patients.

**Registration:**

This Systematic review and Meta-Analysis was registered in Prospero international prospective register of systemic reviews (CRD42020177095) on April 9/2020.

## 1. Introduction

The Coronavirus belongs to large groups of viruses that cause serious health problems affecting the respiratory, gastrointestinal, liver, and central nervous system of humans, livestock, Bats, mice, and other wild animals [1–6]. The infection mainly affects the respiratory system and manifested with fever, dry cough, and difficulty breathing. In the late stages of the infection, the patient may die due to pneumonia and acute respiratory distress syndrome [4, 7–10].

The Severe acute respiratory syndrome (SARS-CoV-2) novel coronavirus was identified in Wuhan, Hubei province of China in December 2019 by the Chinese Center for Disease and Prevention from the throat swab of a patient and the virus is named severe acute respiratory distress COV-2 by WHO which causes Coronaviruses disease 2019 (COVID-19) [11, 12].

The clinical manifestation of the current coronavirus infection is similar to Severe acute respiratory syndrome (SARS-CoV) outbreak that occurred in the Guangdong Province of China by the year 2002–2003 [13–16] and another novel human coronavirus called Middle East Respiratory Syndrome-CoV (MERS-CoV) which was identified in the Middle East and other Arabian regions in 2012 [17–20].

The World Health Organization (WHO) is named the current virus as severe acute respiratory distress COV-2 which causes coronaviruses disease 2019 (COVID-19). The WHO has declared the novel coronavirus (COVID-19) outbreak as a global pandemic on March 11, 2020 [21].

Globally, More than 5 million confirmed cases and 400, 000 deaths were reported by the World Health Organization (WHO) as of June 9, 2020 [22]. The American region accounted for the highest number of cases and deaths which was more than 3 million and 200,000 respectively. The European region accounted for the second-highest confirmed cases and death which were more than 2 million confirmed cases and 183 thousand deaths. The total number of confirmed cases and death in the Eastern Mediterranean region accounted for approximately 660, 000, and 15,000 respectively [22].

The number of laboratory-confirmed cases and deaths in the African region was the lowest for the last couple of months but the rate of spreading in this region is increasing at an alarming rate and expected to be very high in the next couple of months if it continues as this rate. The current report in Ethiopia is very small which is 2500 confirmed cases and 27 deaths but there are many cases in short periods which is more than150 cases per day [22]. It is estimated that the number even may be very high because the diagnosis is limited only in the capital.

The challenge of COVID-19 is very high globally due to a lack of proven treatment and the complexity of its transmission [12, 19, 23–28]. However, it will be more catastrophic for low and middle-income countries because of very poor health care system, high illiteracy and low awareness of the disease and its prevention, lack of skilled health personnel, scarce Intensive Care Unit, a limited number of mechanical ventilators and prevalence of co-morbidities/infection along with malnutrition.

The severity of the disease is depending on several factors. Studies showed that patients with co-morbidities including (Asthma, COPD, Tuberculosis, Pneumonia, Acute respiratory distress syndrome (ARDS), Diabetes mellitus, hypertension, renal disease, hepatic disease, and cardiac disease), history of smoking, and history of substance use, male gender and age greater than 60 years were more likely to die or develop undesirable outcomes [25, 28–35].

The outcomes of patients with coronavirus infection are very variable. Studies also showed that the rate of ICU admission among coronavirus infected patients was higher which ranged from 3% to 100% of confirmed cases [14, 17, 19, 26, 28, 36–39]. Studies also showed that the prevalence of mortality among intensive care patients with coronavirus infection was very high which ranged from 6% to 86% of admitted patients [14, 17, 19, 26, 28, 36–39].

The global rate of ICU admission, the prevalence of mortality, comorbidities, complication, number of cases demanding mechanical ventilator, length of stay and independent risk factors for ICU mortality are very important variables to be determined to reduce patient mortality and morbidity through varies mitigating strategies including but not limited to increasing number of ICU beds, mechanical ventilator, skilled professionals, integrated monitors and reducing possible risk factors. Therefore, the objectives of this systematic review and Meta-Analysis was to provide global evidence on the rates of ICU admission, the prevalence of mortality, comorbidity, complications, and independent risk factors of mortality among patients with COVID-19 admitted in ICU.

## 2. Materials and methods

### 2.1. Protocol and registration

The systematic review and meta-analysis were conducted based on the Preferred Reporting Items for Systematic and Meta-analysis (PRISMA) protocols [40]. This Systematic Review and Meta-Analysis was registered in Prospero international prospective register of systemic reviews (CRD42020177095) on April 9/2020.

### 2.2. Inclusion and exclusion criteria

#### 2.2.1. Inclusion criteria

All observational (case series, cross-sectional, cohort, and case-control) studies reporting rate of ICU admission, the prevalence of mortality, morbidity, complication, and its determinants among ICU admitted patients with coronavirus (SARS-COV, MERS and SARS-COV 2) were included.

#### 2.2.2. Exclusion criteria

Studies that didn’t report the rate of ICU admission, the prevalence of ICU mortality, and risk factors among patients with coronavirus were excluded. Besides, Randomized controlled trials, case-control studies, Systemic reviews, and Case reports were excluded.

### 2.3. Outcomes of interest

#### 2.3.1. Primary outcomes

The primary outcome of interest was rates of ICU admission and mortality among patients admitted with Coronaviruses during SARS, MERS, and COVID-19 pandemic.

#### 2.3.2. Secondary outcomes

Prevalence of morbidity, the prevalence of complication, and its determinants among patients admitted with Coronaviruses during SARS, MERS, and COVID-19 pandemic.

### 2.4. Search strategy

The search strategy was intended to explore all available published and unpublished studies among Coronaviruses infected patients admitted to ICU from December 2002 to May 2020 without language restrictions. A comprehensive initial search was employed in PubMed/Medline, Science direct, and LILACS followed by an analysis of the text words contained in Title/Abstract and indexed terms. A second search was undertaken by combining free text words and indexed terms with Boolean operators. The third search was conducted with the reference lists of all identified reports and articles for additional studies. Finally, an additional and grey literature search was conducted on Google scholars. The PubMed/Medline database was searched with the following terms: SARS[Title/Abstract]) OR (SARS-COV-2[Title/Abstract])) OR (COVID-19[Title/Abstract])) AND (MERS[Title/Abstract])) AND (mortality[Title/Abstract])) OR (morbidity[Title/Abstract])) AND (ICU[Title/Abstract])) OR (hospital[Title/Abstract])) AND (prevalence[Title/Abstract])) AND (risk factors[Title/Abstract])).

### 2.5. Data extraction

The data from each study were extracted with two independent authors with a customized format. The disagreements between the two independent authors were resolved by the other two authors. The extracted data included: Author names, country, date of publication, sample size, the rates of ICU admission, mortality, types of Coronavirus, types of comorbidity, complications, and risk factors. Finally, the data were then imported for analysis in R software version 3.6.1 and STATA 14.

### 2.6. Assessment of methodological quality

Articles identified for retrieval were assessed by two independent Authors for methodological quality before inclusion in the review using a standardized critical appraisal Tool adapted from the Joanna Briggs Institute [45,46] (S1 Table). The disagreements between the Authors appraising the articles were resolved through discussion with the other Two Authors. Articles with average scores greater than fifty percent were included for data extraction.

### 2.7. Data analysis

Data analysis was carried out in R statistical software version 3.6.1 and STATA 14. The pooled rates of ICU admission and prevalence of mortality, comorbidity, complication among corona virus-infected patients were determined with a random effect model as there was substantial heterogeneity between the included studies. The Heterogeneity among the included studies was checked with forest plot, χ2 test, I^2^ test, and the p-values. Subgroup analysis was conducted by Country, type of coronavirus, types of comorbidity, and complications. Publication bias was checked with a funnel plot and the objective diagnostic test was conducted with Egger’s correlation, Begg's regression tests, and Trim and fill method. Furthermore, moderator analysis was carried out to identify the independent predictors of ICU mortality among corona cases. The results were presented based on the Preferred Reporting Items for Systemic Reviews and Meta-Analysis (PRISMA) [40].

### 2.8. Ethics approval and consent to participate

Ethical clearance and approval were obtained from the ethical review board of the College of Health Science and Medicine.

## 3. Results

### 3.1. Selection of studies

**Fig 1:**
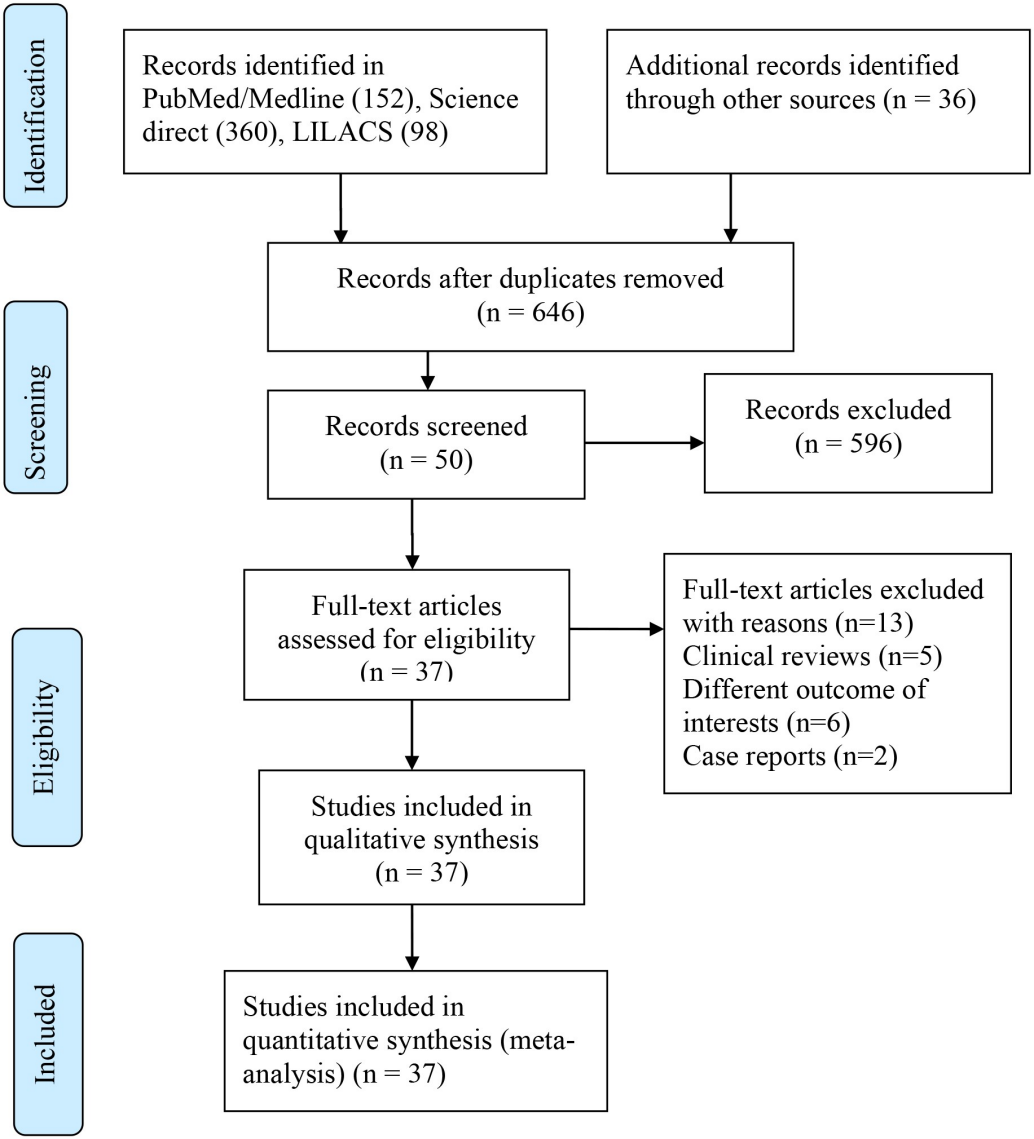
Prisma flow chart.

A total of 646 articles were identified from different databases with an initial search. Fifty articles were selected for evaluation after the successive screening. Thirty-seven Articles with 24983 participants were included in the systematic review and Meta-Analysis while thirteen studies were excluded with reasons (Fig 1).

### 3.2. Characteristics of included studies

Thirty-seven studies conducted on Coronavirus reporting rates of ICU admission and patient outcomes with 24983 participants were included (Table 1). Thirteen studies were excluded with reasons (S1 Table). The methodological quality of included studies was moderate to high quality as depicted with the Joanna Briggs Appraisal tool for observational studies (S2 Table).

**Table 1:** Methodological quality of included studies.

Author(s)	Year	Event	Sample	Country	Types of Coronavirus	Quality Score	Prevalence (95% CI)
Liu et al[41]	2020	7	11	China	SARS-COV-2	8	64(31, 89)
Xu et al[42]	2020	1	2	China	SARS-COV-2	6	50(1, 99)
Arentz et al[37]	2020	11	17	USA	SARS-COV-2	5	65(38, 86)
Bhatraju et al[43]	2020	12	24	USA	SARS-COV-2	5	50(29, 71)
Bialek et al[44]	2020	55	121	USA	SARS-COV-2	5	45(36, 55)
Cao et al[45]	2020	3	4	China	SARS-COV-2	4	75(19, 99)
Chen et al[46]	2020	2	22	China	SARS-COV-2	6	9(1,29)
Chen et al[14]	2020	11	23	China	SARS-COV-2	8	48(27, 69)
Huang et al[47]	2020	6	13	China	SARS-COV-2	6	46(19, 75)
Petrilli et al[48]	2020	116	457	USA	SARS-COV-2	6	25(21, 30)
Richardson et al[49]	2020	18	373	USA	SARS-COV-2	7	5(3, 8)
Simonnet et al[50]	2020	18	124	France	SARS-COV-2	5	15(9, 22)
Wang et al[51]	2020	6	36	China	SARS-COV-2	6	17(6, 33)
Wu et al[52]	2020	44	53	China	SARS-COV-2	6	83(70,72)
Yang et al[28]	2020	32	52	China	SARS-COV-2	6	62(47, 75)
Young et al[6]	2020	1	2	Singapore	SARS-COV-2	6	50(1, 99)
Guan et al[53]	2020	15	1099	China	SARS-COV-2	6	1(1, 2)
Zhou et al[54]	2020	39	50	China	SARS-COV-2	6	78(64, 88)
Lodigiania et al[55]	2020	8	62	Italy	SARS-COV-2	7	13(6, 24)
Kloka et al[56]	2020	41	184	Holland	SARS-COV-2	5	22(16, 29)
Lei et al [57]	2020	7	15	China	SARS-COV-2	6	47(21, 73)
Docherty et al[58]	2020	3001	20133	UK	SARS-COV-2	6	15(14, 15)
Du et al [59]	2020	6	51	China	SARS-COV-2	5	12(4, 24)
Ling et al[60]	2020	8	49	China	SARS-COV-2	5	16(7, 30)
Zangrillo et al [61]	2020	14	61	Italy	SARS-COV-2	4	23(13, 35)
Grasselli et al [62]	2020	405	1591	Italy	SARS-COV-2	6	25(23, 28)
Chan et al[13]	2003	18	39	China	SARS-COV	7	46(30, 63)
Chen et al[12]	2005	21	33	Taiwan	SARS-COV	5	64(45, 80)
Choi et al[15]	2003	32	69	China	SARS-COV	8	46(34, 59)
Lew TW et al[63]	2003	20	46	Singapore	SARS-COV	8	43(29, 59)
Almekhlafie et al[64]	2016	23	27	Saudi Arabia	MERS-CoV	6	85(66, 96)
Al-Hameed et al[18]	2016	5	8	Saudi Arabia	MERS-CoV	6	63(24, 91)
Garbati et al[65]	2016	1	4	Saudi Arabia	MERS-CoV	8	25(1, 81)
Al Ghamdi et al[66]	2016	19	37	Saudi Arabia	MERS-CoV	5	51(34, 68)
Halim et al[26]	2016	14	32	Saudi Arabia	MERS-CoV	7	44(26, 62)
Saad et al[33]	2014	42	49	Saudi Arabia	MERS-CoV	8	86(73, 94)
Arabi YM et al[19]	2014	5	10	Saudi Arabia	MERS-CoV	6	50(19, 81)

Q: question; Y: yes; N: No

Twenty-six of the included studies were conducted on a newly emerged Coronavirus (SARS-CoV-2), COVID-19. Seven studies were conducted during and after the aftermath of the Middle East respiratory syndrome epidemic in the Middle East and other Arabian regions in 2012 while the remaining four studies were conducted during the severe acute respiratory syndrome (SARS-CoV) outbreak in China in 2002.

The included studies were conducted in different regions of the world. Sixteen studies were conducted in China, seven studies in Saudi Arabia, five studies in the United States of America, three studies in Italy, two studies in Singapore, one study in Holland, the United Kingdom, and France.

All of the included studies reported rates of ICU admission and outcomes of patients while staying in ICU. The majority of the included studies reported the presence of comorbidities and complications in ICU such as death, acute respiratory distress syndrome, renal failure, shock, and discharge.

### 3.3. Meta-analysis

#### 3.3.1. Rate of ICU admission

**Fig 2:**
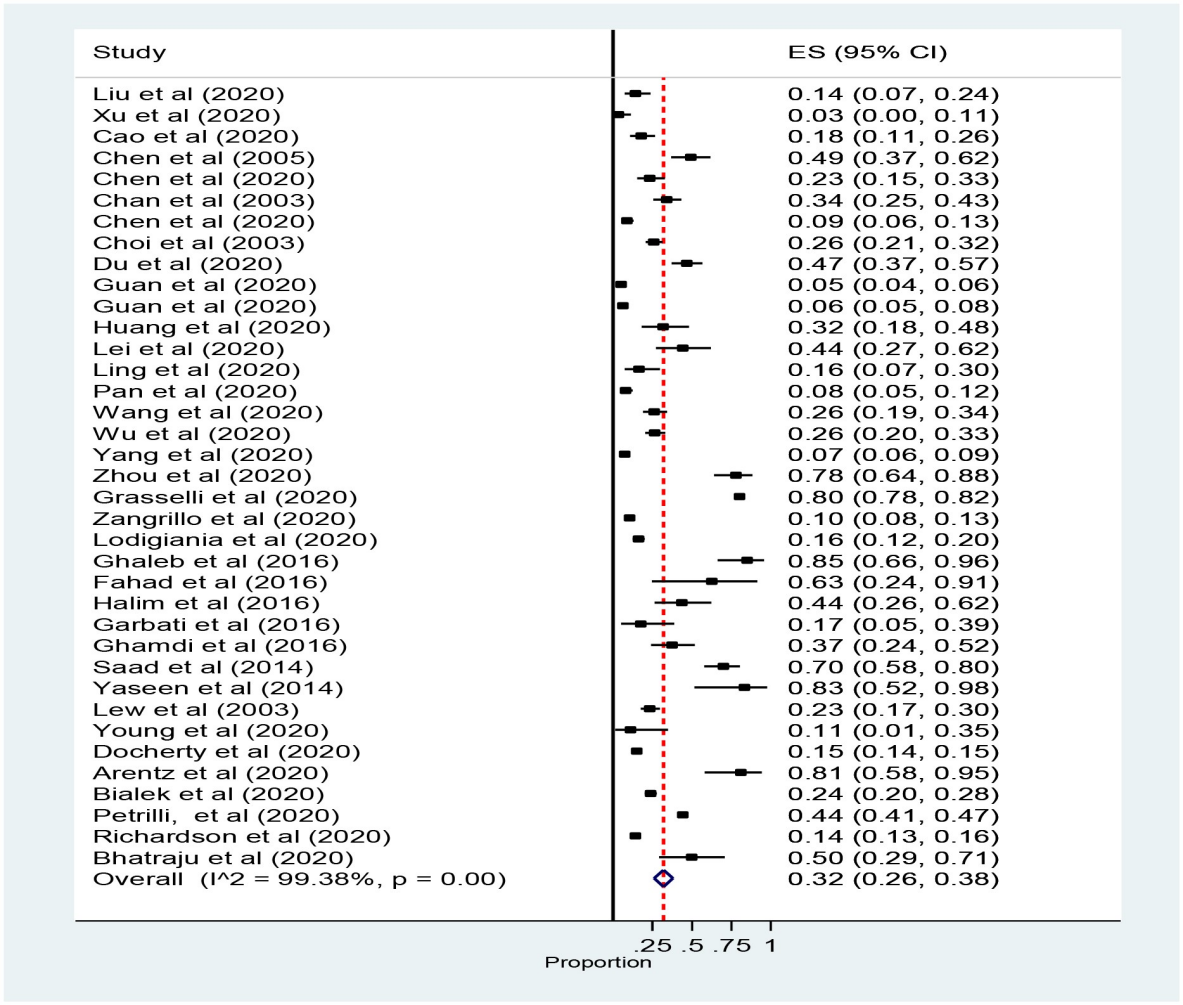
Forest plot for the prevalence of ICU admission patients with coronavirus: The midpoint of each line illustrates the prevalence; the horizontal line indicates the confidence interval, and the diamond shows the pooled prevalence. ICU: Intensive Care Unit.

Thirty-seven studies reported ICU admission were included for Meta-analysis. The number of ICU admission was taken for estimation of pooled prevalence of mortality instead of the total sample size because we wanted to know the number of ICU deaths from those Admitted in ICU. However, the rates of ICU admission were estimated with the total sample size. The pooled rate of ICU admission was 32% (95% CI: 26 to 38, 37 studies and 32, 741 participants) (Fig 2).

**Fig 3:**
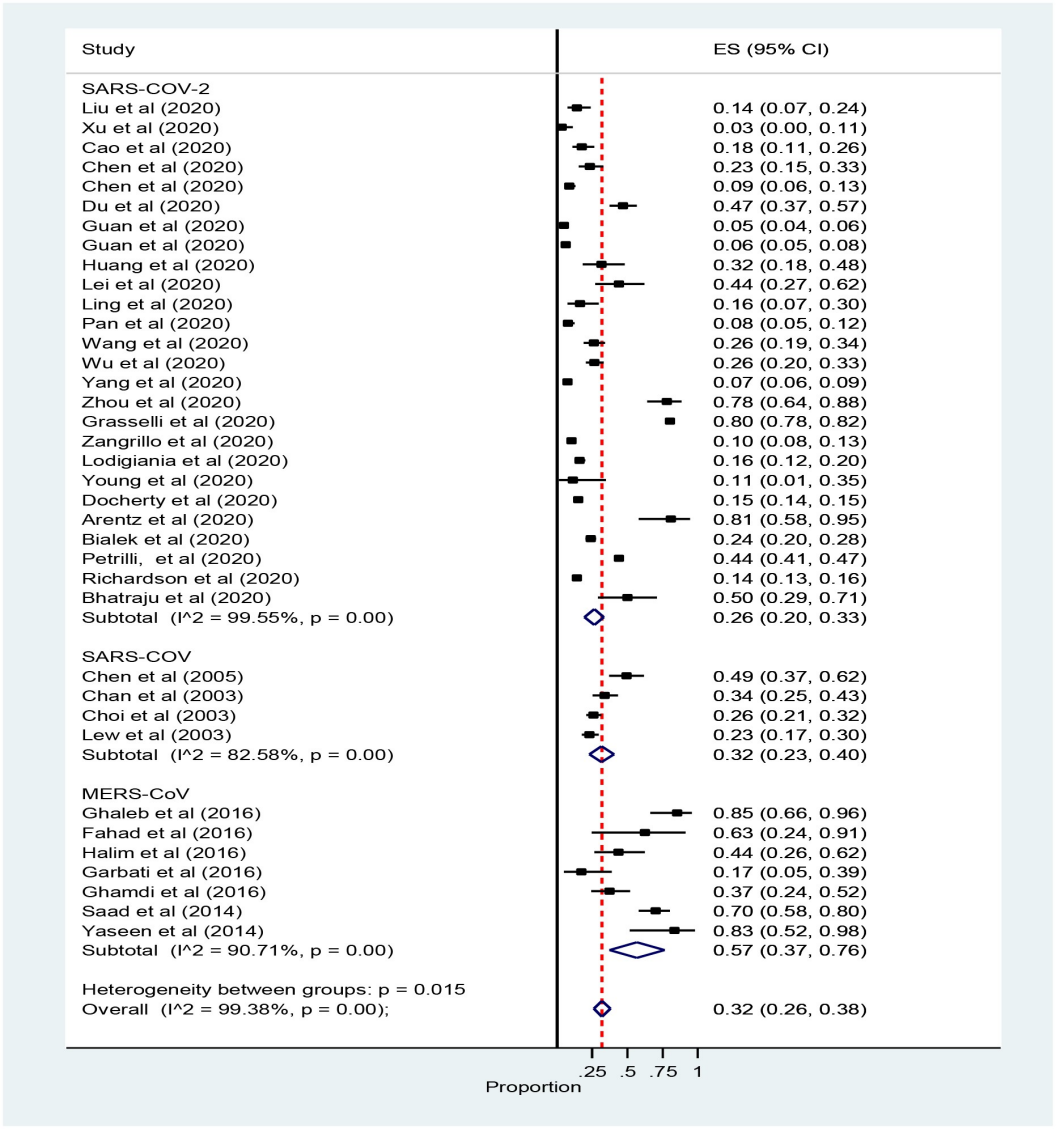
Forest plot for subgroup analysis prevalence of ICU admission patients with coronavirus: The midpoint of each line illustrates the prevalence; the horizontal line indicates the confidence interval, and the diamond shows the pooled prevalence. ICU: Intensive Care Unit.

The finding of the subgroup analysis by types of corona revealed that the rate of ICU admission with SARS-COV, MERS and SARS-COV-2 was 32% (95% CI, 23 to 40), 57% 95% CI, 37 to 76) and 26% 95% CI, 20 to 33) respectively (Fig 3).

#### 3.3.2. Prevalence of ICU mortality

**Fig 4:**
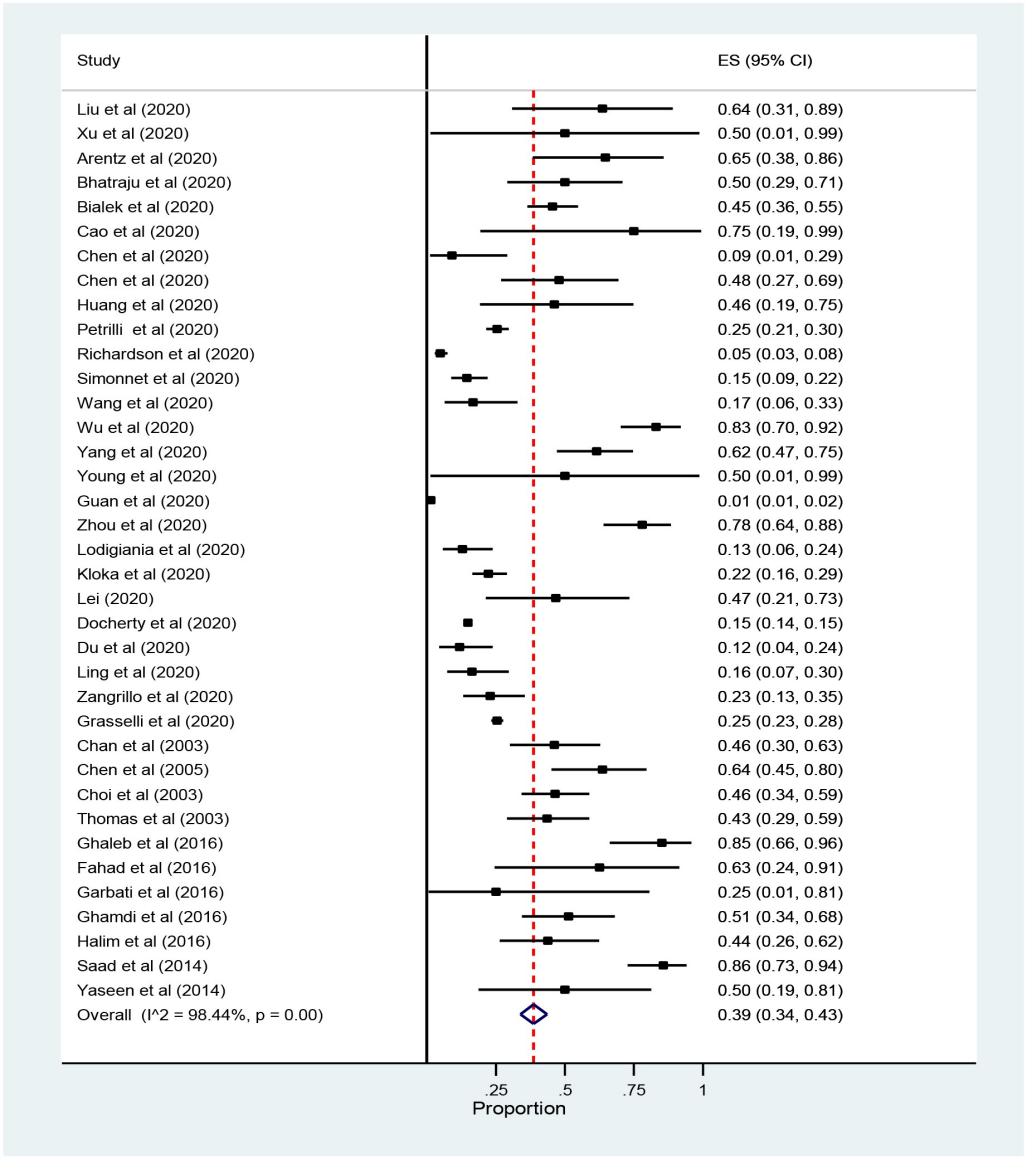
Forest plot for the prevalence of ICU mortality among patients with coronavirus: The midpoint of each line illustrates the prevalence; the horizontal line indicates the confidence interval, and the diamond shows the pooled prevalence. ICU: Intensive Care Unit.

The Meta-Analysis showed that the prevalence of mortality among ICU admitted patients with Coronavirus was 39% (95% CI: 34 to 43, 37 studies and 24, 983 participants) (Fig 4).

**Fig 5:**
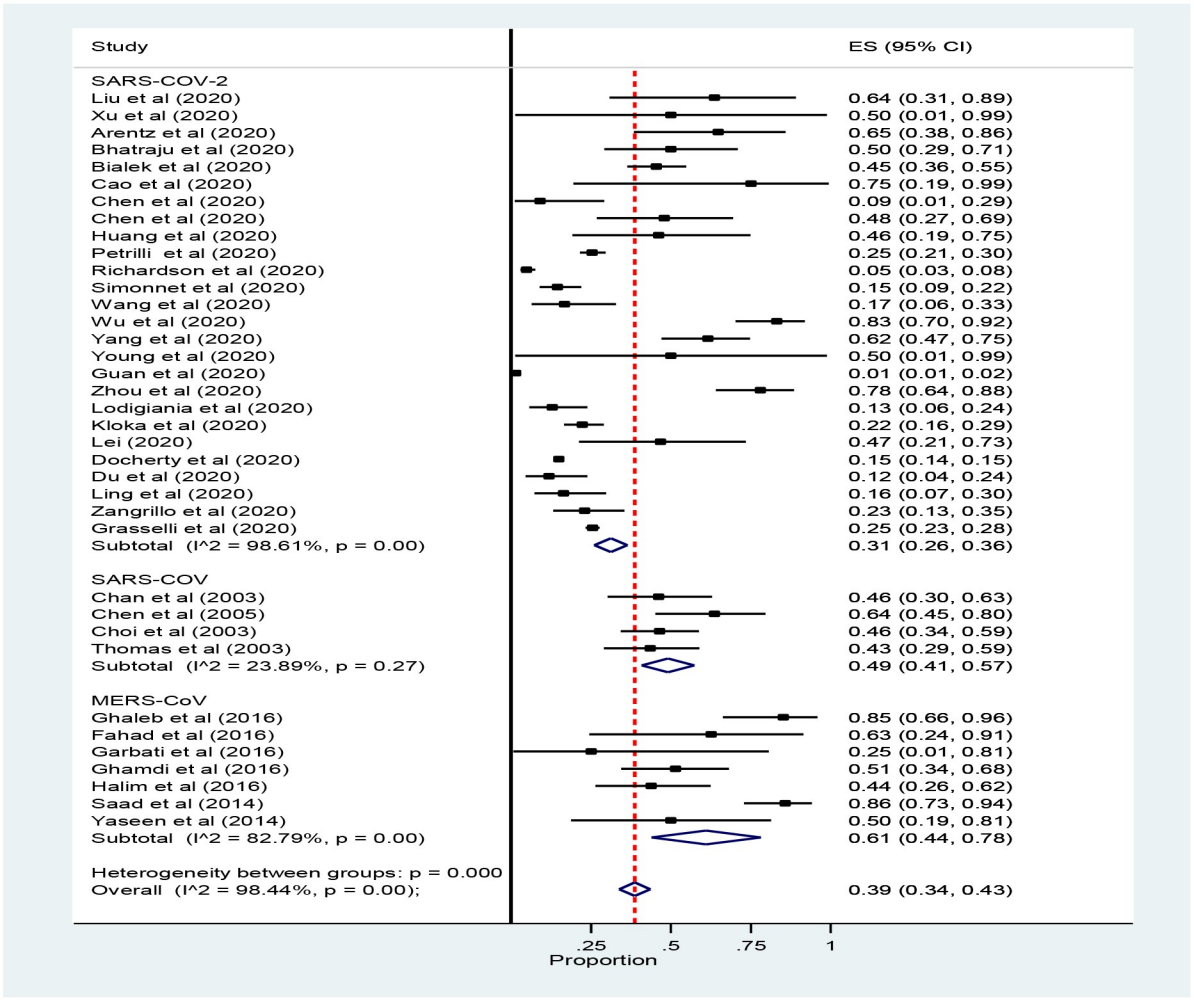
Forest plot for subgroup analysis of the prevalence of ICU mortality among patients with coronavirus: The midpoint of each line illustrates the prevalence; the horizontal line indicates the confidence interval, and the diamond shows the pooled prevalence. ICU: Intensive Care Unit.

The subgroup analysis of the pooled prevalence of mortality among ICU admitted patients with Coronavirus showed that mortality was higher in Saudi Arabia with the Middle East respiratory syndrome 61%(95% CI: 44 to 78) while the prevalence of ICU mortality among patients with the severe acute respiratory syndrome (SARS-CoV-2) was 31% (95% CI: 26to 36) (Fig 5).

The subgroup analysis by country revealed that ICU mortality with COVID-19 was 31% (95% CI: 44 to 78, 25 studies, 24677 participants) where the highest was in China 42% (95% CI: 23 to 61, 13 studies, 1480 participants) followed by USA 36% (95% CI: 18 to 53, 5 studies, 992 participants) (S1 Fig).

#### 3.3.3. Prevalence of comorbidity

**Fig 6:**
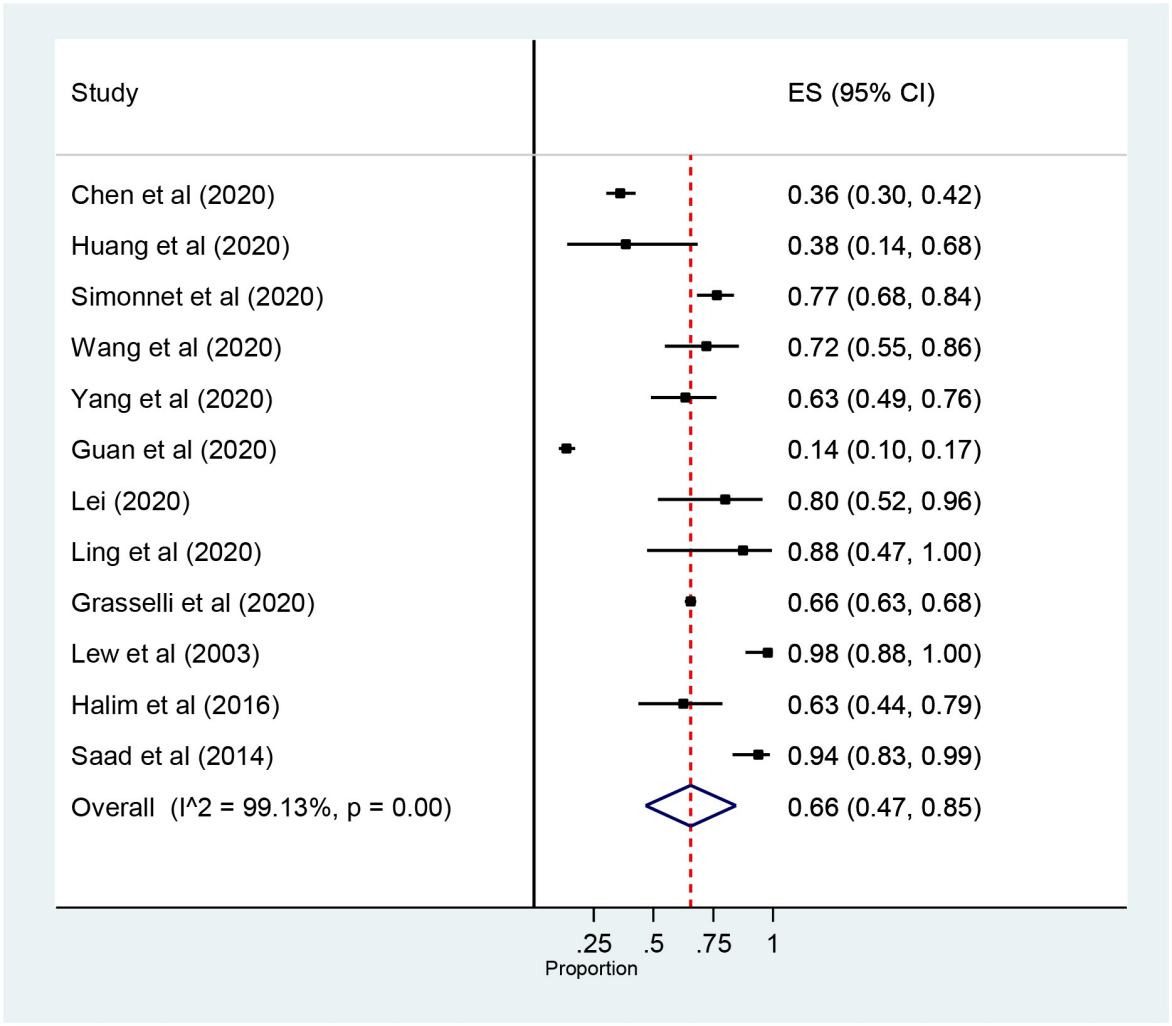
Forest plot for the prevalence of ICU Comorbidity among patients with coronavirus: The midpoint of each line illustrates the prevalence; the horizontal line indicates the confidence interval, and the diamond shows the pooled prevalence. ICU: Intensive Care Unit.

The prevalence of comorbidity among ICU patients with coronavirus was 66% (95% confidence interval (CI): 47 to 85, 12 studies, and 2614 participants) (Fig 6). The Meta-Analysis also revealed that the prevalence of comorbidity among COVID-19 Patients admitted in ICU was 59% (95% confidence interval (CI): 39 to 79, 10 studies and 896 participants) (S2 Fig).

**Fig 7:**
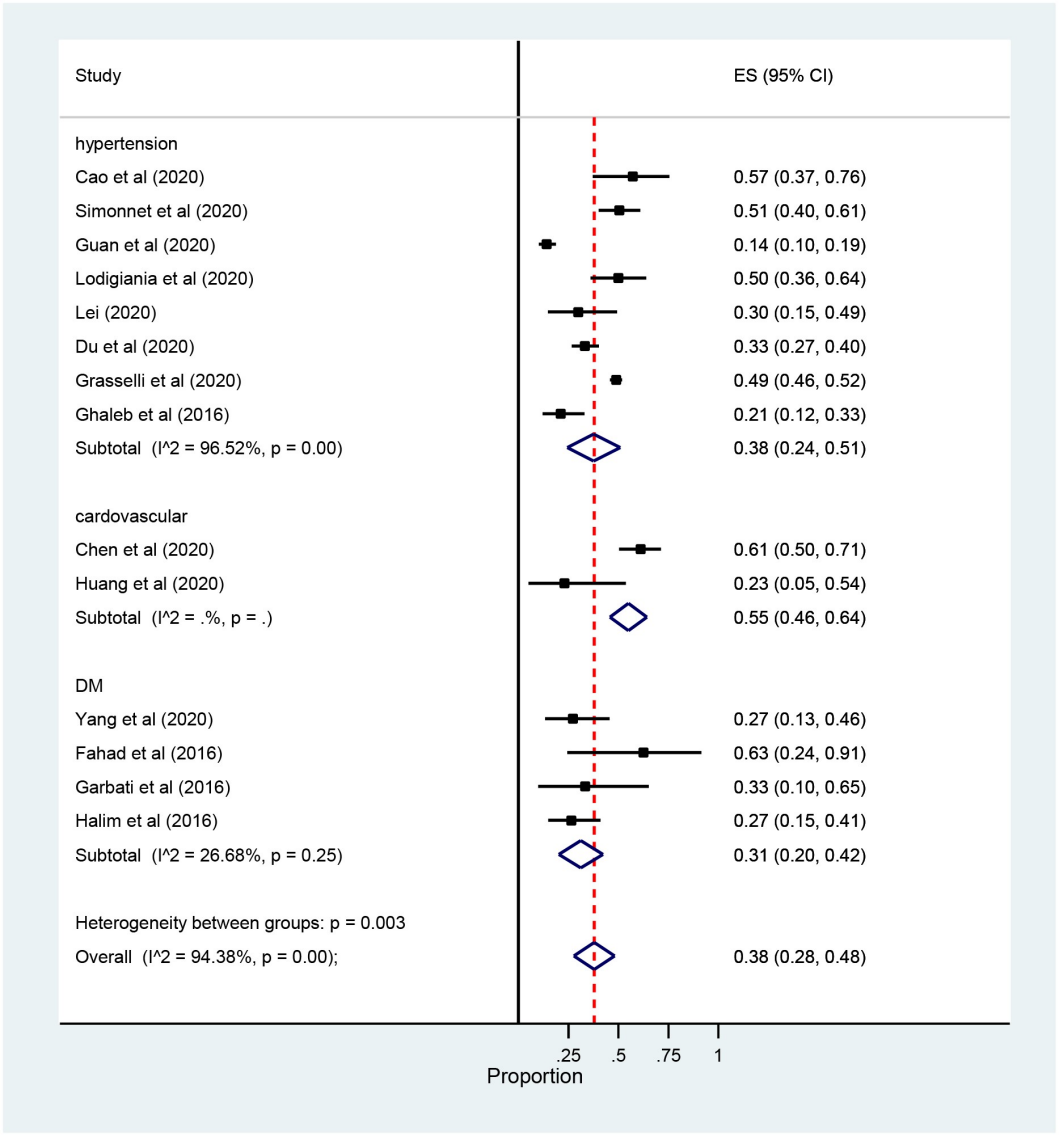
Forest plot for subgroup analysis of the prevalence of ICU Comorbidity among patients with coronavirus: The midpoint of each line illustrates the prevalence; the horizontal line indicates the confidence interval, and the diamond shows the pooled prevalence. ICU: Intensive Care Unit.

The subgroup analysis by the types of comorbidity showed that cardiovascular diseases were the most prevalent 55% (95% confidence interval (CI): 46 to 64) followed by hypertension and Diabetes Mellitus, 38% (95% confidence interval (CI): 26 to 55) and 31% (95% confidence interval (CI): 20 42) respectively (Fig 7).

### 3.4. Prevalence of complications

**Fig 8:**
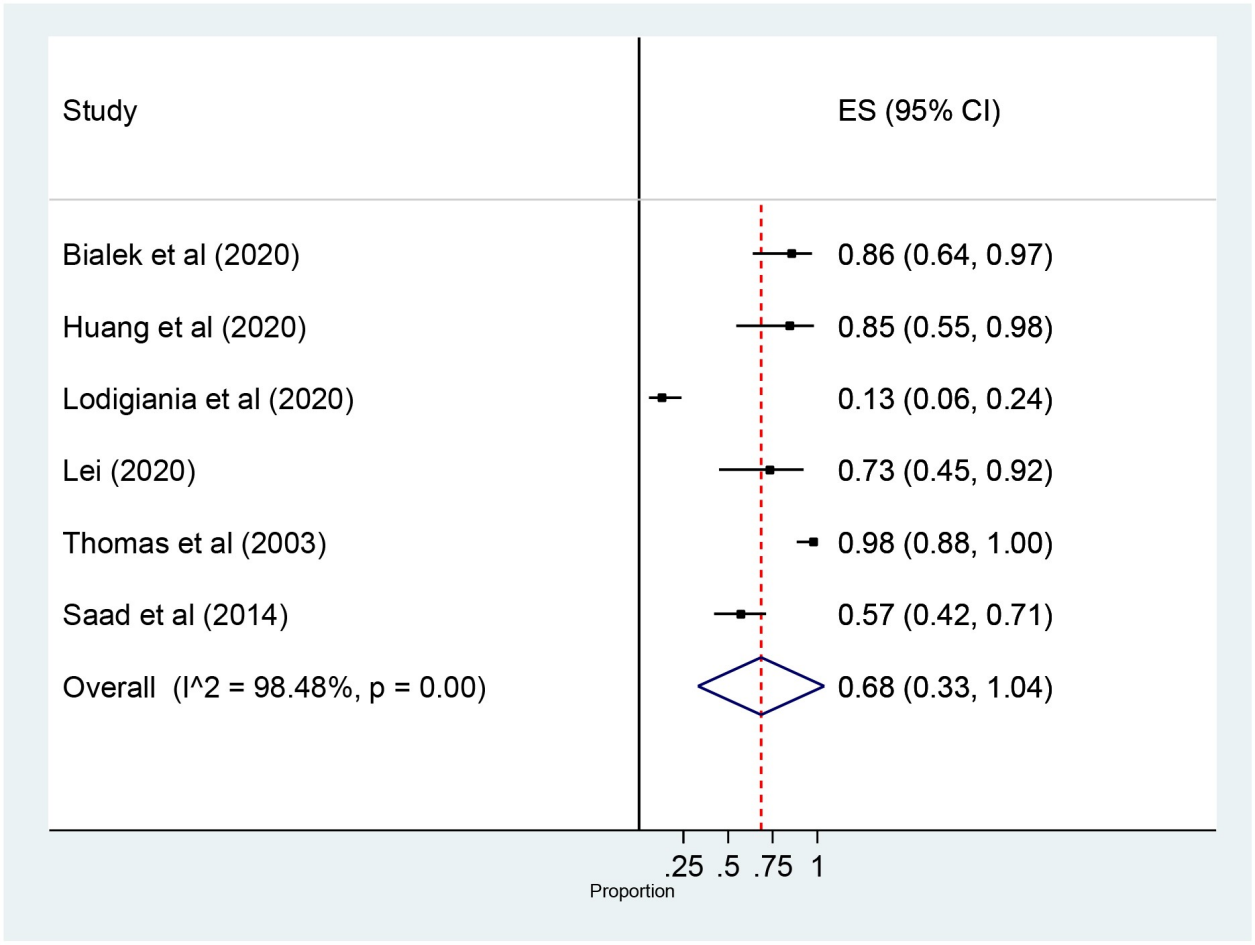
Forest plot for of prevalence of ICU Complication among patients with coronavirus: The midpoint of each line illustrates the prevalence; the horizontal line indicates the confidence interval, and the diamond shows the pooled prevalence. ICU: Intensive Care Unit.

The Meta-Analysis showed that the prevalence of complications among ICU admitted patients with coronavirus was 68% (95% confidence interval (CI): 33 to 104) (Fig 8). The subgroup analysis by types of complication showed that ARDS was the most prevalent complication, 54% (95% confidence interval (CI): 26 to 82) followed by infection and sepsis, 47% (95% confidence interval (CI): 29 to 65) and 37% (95% confidence interval (CI): 26 to 49) respectively (S3 Fig).

### 3.5. Regression analysis

The prevalence of mortality among patients with Coronavirus was greatly affected by several factors including the presence of co-morbidities, history of smoking, history of substance use, male gender, older age groups, ICU admission, nosocomial infection, and others. The regression analysis revealed that patients with ARDS were 2 times more likely to die as compared to those who didn’t develop ARDS, RR = 2.08 (95% confidence interval(CI): 1.48 to 2.93). The risk of mortality among patients who are older than 50 years increased by 13%, RR = 1.87(95% confidence interval (CI): 1.35 to 2.58). The presence of any comorbidity increased the risk of death by 39%, RR = 1.61(95% confidence interval (CI): 1.24 to 2.09) (S4 Fig).

### 3.6. Sensitivity analysis and publication bias

Sensitivity analysis was conducted to identify the most influential study on the pooled summary effect and we didn’t find significant influencing the summary effect.

**Fig 9:**
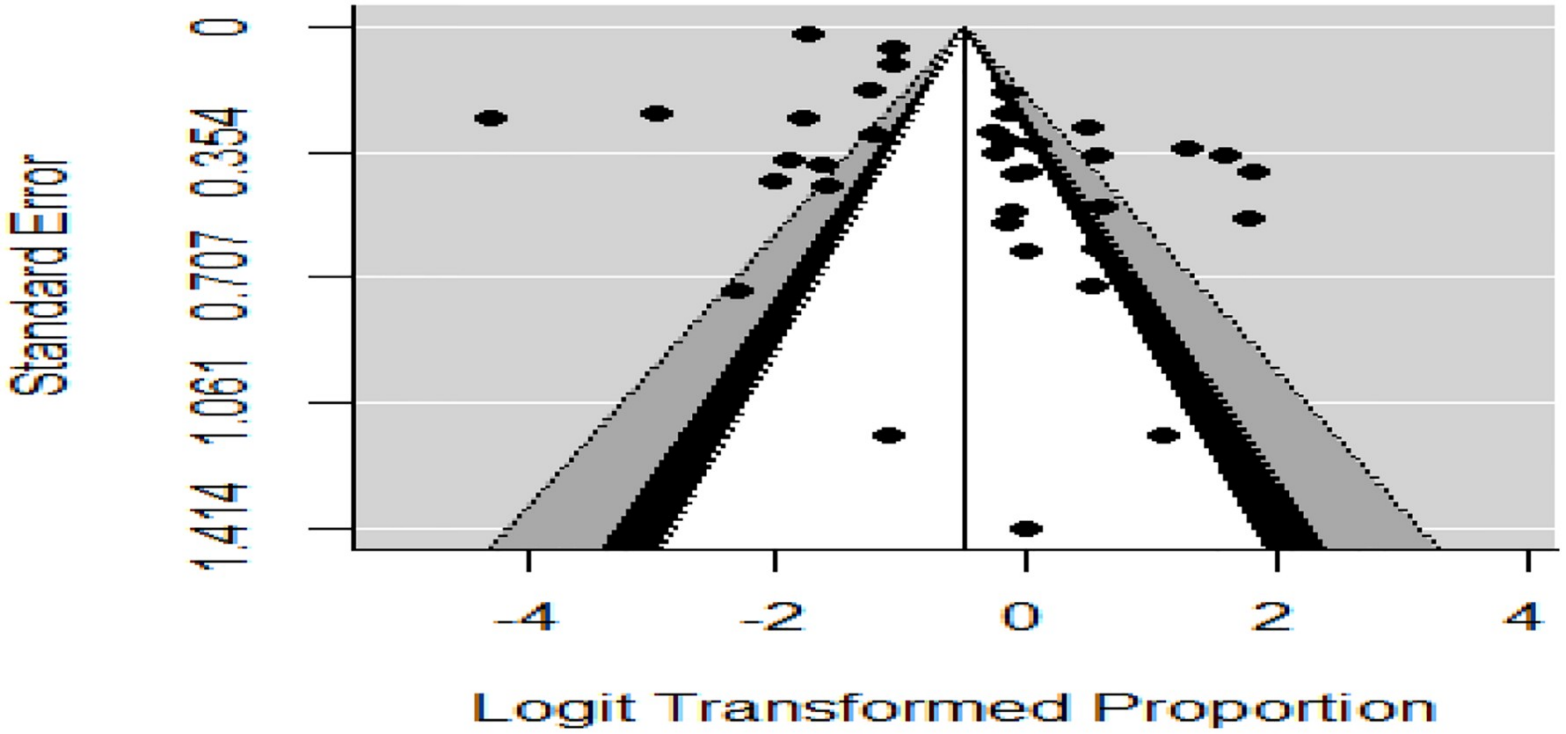
Funnel plot to assess publication bias. The vertical line indicates the effect size whereas the diagonal line indicates the precision of individual studies with a 95% confidence interval.

Publication bias was investigated with funnel plot asymmetry and egger’s regression and Begg’s rank correlation were run to investigate publication bias objectively. The funnel plot didn’t show significant publication bias. Neither egger’s regression nor Begg’s rank correlation showed significant publication bias (P-value < 0.1464) (Fig 9).

## 4. Discussion

The Meta-Analysis revealed that more than one-third of patients with coronavirus infection were admitted to ICU globally. The subgroup analysis showed that the rate of ICU admission was very high in patients with the Middle East respiratory syndrome (MERS-CoV), 57% (95% CI: 37to 76) as compared to severe acute respiratory syndrome (SARS-CoV-2 and SARS-CoV), 26% (95% CI: 20 to 33) and 32% (95% CI: 23 to 40) respectively. Currently, the total confirmed cases and the death of patients with the SARS-CoV-2 virus is unpredictably high as compared to the previous two outbreaks [13–15, 19, 20, 63, 64, 66–68]. The lower rate of ICU admission in patients with COVID-19 in this systematic review and Meta-Analysis might be due to a small number of studies assessing rates of admission compared to the number of cases and also the majority of studies were case series with small sample size.

This systematic review and Meta-Analysis revealed that the prevalence of mortality among Coronavirus confirmed cases admitted in ICU were, 39% (95% CI: 34 to 43). This finding is interpreted as there is one mortality for every three cases of admission. This finding is in line with individual studies conducted among Coronavirus confirmed cases since the first outbreak in 2002, China [13–15, 19, 20, 63, 64, 66–68]. The possible explanation for a high number of deaths in ICU may be explained in terms of a limited number of mechanical ventilators, adequate laboratory investigation, integrated patient monitors, presence of co-morbidities, hospital-acquired infections, and some others.

The subgroup analysis showed that the prevalence of mortality among COVID-19 patients admitted in ICU was very higher, 31% (95% CI: 26 to 36). But, it is relatively low as compared to MERS-CoV and SARS-CoV, 61% (95% CI: 44 to 78), and 49% (95% CI: 41 to 57) respectively. The possible explanation for the lower prevalence of mortality among COVID-19 patients might be due to better ICU supportive management, skilled ICU professionals, integrated patient monitors, and lessons from previous outbreaks in handling ICU cases.

The pooled prevalence of comorbidity among patients with coronavirus was as high as sixty percent. The subgroup analysis revealed that the prevalence of comorbidity among COVID-19 patients was 59% (95% confidence interval (CI): 39 to 79) which is consistent with findings of subgroup analysis of SARS-COV, MERS-COV, and individual included studies. The regression analysis revealed that presences of comorbidity, male gender, age greater than 50 years, and ARDS were independent predictors of mortality among patients admitted in ICU with coronaviruses.

### 4.1. Quality of evidence

The systematic review and meta-analysis included plenty of studies with adequate sample size. The methodological quality of included studies was moderate to high quality as depicted with Joanna Briggs Institute assessment tool for meta-analysis of observational studies. However, substantial heterogeneity associated with dissimilarities of included studies in sample size, design, and location could affect the allover quality of evidence.

### 4.2. Limitation of the study

The review incorporated plenty of studies with a large number of participants but the majority of studies included in this review didn’t report data on comorbidity and risk factors to investigate the independent predictors. Besides, there were a limited number of studies in some countries and it would be difficult to provide conclusive evidence with results pooled from fewer studies.

### 4.3. Implication for practice

Body of evidence revealed that rate of ICU admission; the prevalence of mortality; morbidity and complications were very high among patients with COVID-1. These could be a huge impact particularly for low and middle-income countries with a limited number of ICU beds, mechanical ventilator, integrated patient monitor, skilled professionals combined with malnutrition, and communicable disease. Therefore, a mitigating strategy is required by different stakeholders to combat the catastrophic impacts of COVID-19 pandemic through creating awareness about preventive measures, implementing ICU protocols for supportive management, management of comorbidities, and prevention of complications.

### 4.4. The implication for further research

The meta-analysis revealed that the prevalence of mortality among COVD-19 in ICU was very high and the major independent predictors of mortality were identified. However, the included studies were too heterogeneous, and cross-sectional studies also don’t show a temporal relationship between mortality and its determinants. Therefore, further observational and randomized controlled trials are in demand for a specific group of patients by stratifying the possible independent predictors.

## 5. Conclusion

The systematic review and Meta-Analysis revealed that approximately one-third of patients admitted to ICU with severe Coronavirus disease. The systematic review also showed that more than thirty percent of patients admitted in ICU with a severe form of COVID-19 for better care died which warns the health care stakeholders to give attention to intensive care patients admitted with COVID-19 through accessing mechanical ventilators, integrated patient monitors, skilled ICU staffs, creation of awareness about infection prevention and more others. Besides, the prevalence of mortality had a strong relation with comorbidity, age, gender, and complication.

## Supporting information

S1 Table Description of excluded studies with reasons. (DOCX)

S2 Table Methodological quality of included studies. (DOCX)

S1 Fig Forest plot for subgroup analysis of prevalence of ICU mortality by country: The midpoint of each line illustrates the prevalence; the horizontal line indicates the confidence interval, and the diamond shows the pooled prevalence. ICU: Intensive Care Unit.
(DOCX)

S2 Fig Forest plot for subgroup analysis of prevalence of ICU comorbidity by types of coronavirus: The midpoint of each line illustrates the prevalence; the horizontal line indicates the confidence interval, and the diamond shows the pooled prevalence. ICU: Intensive Care Unit.
(DOCX)

S3 Fig Forest plot for subgroup analysis of prevalence of ICU Complication among patients with coronavirus: The midpoint of each line illustrates the prevalence; the horizontal line indicates the confidence interval, and the diamond shows the pooled prevalence. ICU: Intensive Care Unit.
(DOCX)

S4 Fig Forest plot showing pooled odds ratio (log scale) of the associations between Intensive Care Unit mortality and its determinants (A: Co-morbidities; B: Age greater than 50 years; C: Gender D: ARDS). (DOCX)

S1 Checklist PRISMA checklist. (DOC)

## References

[1] Kanwar A, Selvaraju S, Esper F. Human coronavirus-HKU1 infection among adults in Cleveland, Ohio Open forum infectious diseases: Oxford University Press, 2017. 10.1093/ofid/ofx052 PMC5466428 28616442

[2] Lau SK, Chan JF. Coronaviruses: emerging and re-emerging pathogens in humans and animals. BioMed Central, 2015. 10.1186/s12985-015-0432-z PMC4687117 26690088

[3] Wang L-F, Shi Z, Zhang S, et al Review of bats and SARS. Emerging infectious diseases. 2006; 12: 1834 10.3201/eid1212.060401 17326933 PMC3291347

[4] Weiss SR, Navas-Martin S. Coronavirus pathogenesis, and the emerging pathogen severe acute respiratory syndrome coronavirus. Microbiol Mol Biol Rev. 2005; 69: 635–64. 10.1128/MMBR.69.4.635-664.2005 16339739 PMC1306801

[5] Wu D, Wu T, Liu Q, et al The SARS-CoV-2 outbreak: what we know. International Journal of Infectious Diseases. 2020. 10.1016/j.ijid.2020.03.004 PMC7102543 32171952

[6] Young BE, Ong SWX, Kalimuddin S, et al Epidemiologic features and clinical course of patients infected with SARS-CoV-2 in Singapore. Jama. 2020; 323: 1488–94. 10.1001/jama.2020.3204 PMC7054855 32125362

[7] Adhikari SP, Meng S, Wu Y-J, et al Epidemiology, causes, clinical manifestation and diagnosis, prevention and control of coronavirus disease (COVID-19) during the early outbreak period: a scoping review. Infectious diseases of poverty. 2020; 9: 1–12. 32183901 10.1186/s40249-020-00646-x PMC7079521

[8] Cummings MJ, Baldwin MR, Abrams D, et al Epidemiology, clinical course, and outcomes of critically ill adults with COVID-19 in New York City: a prospective cohort study. The Lancet. 2020. 10.1016/S0140-6736(20)31189-2 PMC7237188 32442528

[9] Jain V, Yuan J-M. Systematic review and meta-analysis of predictive symptoms and comorbidities for severe COVID-19 infection. medRxiv. 2020. 10.1007/s00038-020-01390-7 PMC7246302 32451563

[10] Novel CPERE. The epidemiological characteristics of an outbreak of 2019 novel coronavirus diseases (COVID-19) in China. Zhonghua Liu xing bing xue za zhi = Zhonghua liuxingbingxue zazhi. 2020; 41: 145. 10.3760/cma.j.issn.0254-6450.2020.02.003 32064853

[11] World Health Organization: Coronavirus disease 2019 (COVID-19) Situation Report –72. https://www.who.int/docs/default-source/coronaviruse/situation-reports/20200401-sitrep-72-covid-19.pdf?sfvrsn=3dd8971b_2

[12] Chen N, Zhou M, Dong X, et al Epidemiological and clinical characteristics of 99 cases of 2019 novel coronavirus pneumonia in Wuhan, China: a descriptive study. The Lancet. 2020; 395: 507–13. 10.1016/S0140-6736(20)30211-7 PMC7135076 32007143

[13] Chan J, Ng C, Chan Y, et al Short term outcome and risk factors for adverse clinical outcomes in adults with the severe acute respiratory syndrome (SARS). Thorax. 2003; 58: 686–89. 10.1136/thorax.58.8.686 12885985 PMC1746764

[14] Chen C-Y, Lee C-H, Liu C-Y, et al Clinical features and outcomes of the severe acute respiratory syndrome and predictive factors for acute respiratory distress syndrome. Journal of the Chinese Medical Association. 2005; 68: 4–10. 10.1016/S1726-4901(09)70124-8 15742856 PMC7129615

[15] Choi KW, Chau TN, Tsang O, et al Outcomes and prognostic factors in 267 patients with the severe acute respiratory syndrome in Hong Kong. Annals of internal medicine. 2003; 139: 715–23. 10.7326/0003-4819-139-9-200311040-00005 14597455

[16] Joynt GM, Yap H. SARS in the intensive care unit. Current infectious disease reports. 2004; 6: 228 10.1007/s11908-004-0013-6 15142487 PMC7089324

[17] Al-Dorzi HM, Aldawood AS, Khan R, et al The critical care response to a hospital outbreak of Middle East respiratory syndrome coronavirus (MERS-CoV) infection: an observational study. Annals of intensive care. 2016; 6: 101 10.1186/s13613-016-0203-z 27778310 PMC5078123

[18] Al-Hameed F, Wahla AS, Siddiqui S, et al Characteristics and outcomes of Middle East respiratory syndrome coronavirus patients admitted to an intensive care unit in Jeddah, Saudi Arabia. Journal of intensive care medicine. 2016; 31: 344–48. 10.1177/0885066615579858 25862629

[19] Arabi YM, Arifi AA, Balkhy HH, et al Clinical course and outcomes of critically ill patients with Middle East respiratory syndrome coronavirus infection. Annals of internal medicine. 2014; 160: 389–97. 10.7326/M13-2486 24474051

[20] Assiri A, McGeer A, Perl TM, et al Hospital outbreak of Middle East respiratory syndrome coronavirus. New England Journal of Medicine. 2013; 369: 407–16. 10.1056/NEJMoa1306742 23782161 PMC4029105

[21] World Health Organization: Coronavirus disease 2019 (COVID-19) Situation Report –52. https://www.who.int/docs/default-source/coronaviruse/situation-reports/20200312-sitrep-52-covid-19.pdf?sfvrsn=e2bfc9c0_4

[22] World Health Organization: Coronavirus disease 2019 (COVID-19) Situation Report –141. https://www.who.int/docs/default-source/coronaviruse/situation-reports/20200609-covid-19-sitrep-141.pdf?sfvrsn=72fa1b16_2

[23] Al-Dorzi HM, Aldawood AS, Khan R, et al The critical care response to a hospital outbreak of Middle East respiratory syndrome coronavirus (MERS-CoV) infection: an observational study. Ann Intensive Care. 2016; 6: 101 10.1186/s13613-016-0203-z 27778310 PMC5078123

[24] Al-Dorzi HM, Alsolamy S, Arabi YM. Critically ill patients with Middle East respiratory syndrome coronavirus infection. Critical Care. 2016; 20: 65 10.1186/s13054-016-1234-4 26984370 PMC4794852

[25] Arentz M, Yim E, Klaff L, et al Characteristics and outcomes of 21 critically ill patients with COVID-19 in Washington State. Jama. 2020. 10.1001/jama.2020.4326 PMC7082763 32191259

[26] Halim AA, Alsayed B, Embarak S, et al Clinical characteristics and outcome of ICU admitted MERS corona, virus-infected patients. Egyptian Journal of Chest Diseases and Tuberculosis. 2016; 65: 81–87. 10.1016/j.ejcdt.2015.11.011 32288128 PMC7132710

[27] Lai CC, Shih TP, Ko WC, et al Severe acute respiratory syndrome coronavirus 2 (SARS-CoV-2) and coronavirus disease-2019 (COVID-19): The epidemic and the challenges. Int J Antimicrob Agents. 2020; 55: 105924. 10.1016/j.ijantimicag.2020.105924 PMC7127800 32081636

[28] Yang X, Yu Y, Xu J, et al Clinical course and outcomes of critically ill patients with SARS-CoV-2 pneumonia in Wuhan, China: a single-centered, retrospective, observational study. The Lancet Respiratory Medicine. 2020. 10.1016/S2213-2600(20)30079-5 PMC7102538 32105632

[29] Assiri A, McGeer A, Perl TM, et al Hospital outbreak of Middle East respiratory syndrome coronavirus. N Engl J Med. 2013; 369: 407–16. 10.1056/NEJMoa1306742 23782161 PMC4029105

[30] Liu R, Ming X, Zhu H, et al. Association of Cardiovascular Manifestations with In-hospital Outcomes in Patients with COVID-19: A Hospital Staff Data. medRxiv. 2020.

[31] Liu W, Tao Z-W, Lei W, et al Analysis of factors associated with disease outcomes in hospitalized patients with 2019 novel coronavirus disease. Chinese medical journal. 2020. 10.1097/CM9.0000000000000775 PMC7147279 32118640

[32] Rasmussen SA, Smulian JC, Lednicky JA, et al Coronavirus Disease 2019 (COVID-19) and Pregnancy: What obstetricians need to know. American journal of obstetrics and gynecology. 2020. 10.1016/j.ajog.2020.02.017 PMC7093856 32105680

[33] Saad M, Omrani AS, Baig K, et al Clinical aspects and outcomes of 70 patients with Middle East respiratory syndrome coronavirus infection: a single-center experience in Saudi Arabia. International Journal of Infectious Diseases. 2014; 29: 301–06. 10.1016/j.ijid.2014.09.003 25303830 PMC7110769

[34] Wang D, Hu B, Hu C, et al Clinical Characteristics of 138 Hospitalized Patients With 2019 Novel Coronavirus-Infected Pneumonia in Wuhan, China. JAMA. 2020. 10.1001/jama.2020.1585 PMC7042881 32031570

[35] Yang J, Zheng Y, Gou X, et al Prevalence of comorbidities in the novel Wuhan coronavirus (COVID-19) infection: a systematic review and meta-analysis. International Journal of Infectious Diseases. 2020. 10.1016/j.ijid.2020.03.017 PMC7194638 32173574

[36] Arabi YM, Murthy S, Webb S. COVID-19: a novel coronavirus and a novel challenge for critical care. Intensive care medicine. 2020: 1–4. 10.1007/s00134-020-05955-1 PMC7080134 32125458

[37] Arentz M, Yim E, Klaff L, et al Characteristics and outcomes of 21 critically ill patients with COVID-19 in Washington State. Jama. 2020; 323: 1612–14. 10.1001/jama.2020.4326 PMC7082763 32191259

[38] Chen T, Wu D, Chen H, et al Clinical characteristics of 113 deceased patients with coronavirus disease 2019: a retrospective study. BMJ. 2020; 368. 10.1136/bmj.m1091 PMC7190011 32217556

[39] Rodriguez-Morales AJ, Cardona-Ospina JA, Gutiérrez-Ocampo E, et al Clinical, laboratory, and imaging features of COVID-19: A systematic review and meta-analysis. Travel medicine and infectious disease. 2020: 101623. 10.1016/j.tmaid.2020.101623 PMC7102608 32179124

[40] Moher D, Liberati A, Tetzlaff J, et al Preferred reporting items for systematic reviews and meta-analyses: the PRISMA statement. PLoS med. 2009; 6: e1000097 10.1371/journal.pmed.1000097 19621072 PMC2707599

[41] Liu W, Tao Z-W, Wang L, et al Analysis of factors associated with disease outcomes in hospitalized patients with 2019 novel coronavirus disease. Chinese medical journal. 2020. 10.1097/CM9.0000000000000775 PMC7147279 32118640

[42] Xu X-W, Wu X-X, Jiang X-G, et al Clinical findings in a group of patients infected with the 2019 novel coronavirus (SARS-Cov-2) outside of Wuhan, China: retrospective case series. BMJ. 2020; 368. 10.1136/bmj.m606 PMC7224340 32075786

[43] Bhatraju PK, Ghassemieh BJ, Nichols M, et al Covid-19 in critically ill patients in the Seattle region—case series. New England Journal of Medicine. 2020; 382: 2012–22. 10.1056/NEJMoa2004500 32227758 PMC7143164

[44] COVID C, Team R. Severe outcomes among patients with coronavirus disease 2019 (COVID-19)—United States, February 12–March 16, 2020. MMWR Morb Mortal Wkly Rep. 2020; 69: 343–46. 10.15585/mmwr.mm6912e2 32214079 PMC7725513

[45] Cao J, Hu X, Cheng W, et al Clinical features and short-term outcomes of 18 patients with coronavirus disease 2019 in the intensive care unit. Intensive care medicine. 2020: 1–3. 10.1007/s00134-020-05987-7 PMC7079866 32123993

[46] Chen J, Qi T, Liu L, et al Clinical progression of patients with COVID-19 in Shanghai, China. Journal of Infection. 2020. 10.1016/j.jinf.2020.03.004 PMC7102530 32171869

[47] Huang C, Wang Y, Li X, et al Clinical features of patients infected with 2019 novel coronavirus in Wuhan, China. The lancet. 2020; 395: 497–506. 10.1016/S0140-6736(20)30183-5 PMC7159299 31986264

[48] Petrilli CM, Jones SA, Yang J, et al Factors associated with hospitalization and critical illness among 4,103 patients with COVID-19 disease in New York City. MedRxiv. 2020.

[49] Richardson S, Hirsch JS, Narasimhan M, et al Presenting characteristics, comorbidities, and outcomes among 5700 patients hospitalized with COVID-19 in the New York City area. Jama. 2020. 10.1001/jama.2020.6775 PMC7177629 32320003

[50] Simonnet A, Chetboun M, Poissy J, et al High prevalence of obesity in severe acute respiratory syndrome coronavirus‐2 (SARS‐CoV‐2) requiring invasive mechanical ventilation. Obesity. 2020. 10.1002/oby.22831 PMC7262326 32271993

[51] Wang D, Hu B, Hu C, et al Clinical characteristics of 138 hospitalized patients with 2019 novel coronavirus–infected pneumonia in Wuhan, China. Jama. 2020; 323: 1061–69. 10.1001/jama.2020.1585 PMC7042881 32031570

[52] Wu C, Chen X, Cai Y, et al Risk factors associated with acute respiratory distress syndrome and death in patients with coronavirus disease 2019 pneumonia in Wuhan, China. JAMA internal medicine. 2020. 10.1001/jamainternmed.2020.0994 PMC7070509 32167524

[53] Guan W-j, Liang W-h, Zhao Y, et al Comorbidity and its impact on 1590 patients with Covid-19 in China: A Nationwide Analysis. European Respiratory Journal. 2020; 55. 10.1183/13993003.00547-2020 PMC7098485 32217650

[54] Zhou F, Yu T, Du R, et al Clinical course and risk factors for mortality of adult inpatients with COVID-19 in Wuhan, China: a retrospective cohort study. The lancet. 2020. 10.1016/S0140-6736(20)30566-3 PMC7270627 32171076

[55] Lodigiani C, Iapichino G, Carenzo L, et al Venous and arterial thromboembolic complications in COVID-19 patients admitted to an academic hospital in Milan, Italy. Thrombosis research. 2020. 10.1016/j.thromres.2020.04.024 PMC7177070 32353746

[56] Klok FA, Kruip M, Van Der Meer N, et al Confirmation of the high cumulative incidence of thrombotic complications in critically ill ICU patients with COVID-19: an updated analysis. Thrombosis Research. 2020. 10.1016/j.thromres.2020.04.041 PMC7192101 32381264

[57] Lei S, Jiang F, Su W, et al Clinical characteristics and outcomes of patients undergoing surgeries during the incubation period of COVID-19 infection. clinical medicine. 2020: 100331. 10.1016/j.eclinm.2020.100331 PMC7128617 32292899

[58] Docherty AB, Harrison EM, Green CA, et al Features of 20 133 UK patients in hospital with COVID-19 using the ISARIC WHO Clinical Characterisation Protocol: a prospective observational cohort study. BMJ. 2020; 369. 10.1136/bmj.m1985 PMC7243036 32444460

[59] Du R-H, Liu L-M, Yin W, et al Hospitalization and critical care of 109 decedents with COVID-19 pneumonia in Wuhan, China. Annals of the American Thoracic Society. 2020. 10.1513/AnnalsATS.202003-225OC PMC7328178 32255382

[60] Ling L, So C, Shum HP, et al Critically ill patients with COVID-19 in Hong Kong: a multicentre retrospective observational cohort study. Crit Care Resusc. 2020; 6. 10.51893/2020.2.oa1 PMC10692444 32248675

[61] Zangrillo A, Beretta L, Scandroglio AM, et al Characteristics, treatment, outcomes, and cause of death of invasively ventilated patients with COVID-19 ARDS in Milan, Italy. Crit Care Resusc. 2020. 10.1016/S1441-2772(23)00387-3 PMC10692521 32900326

[62] Grasselli G, Zangrillo A, Zanella A, et al Baseline characteristics and outcomes of 1591 patients infected with SARS-CoV-2 admitted to ICUs of the Lombardy Region, Italy. Jama. 2020; 323: 1574–81. 10.1001/jama.2020.5394 PMC7136855 32250385

[63] Lew TW, Kwek T-K, Tai D, et al Acute respiratory distress syndrome in critically ill patients with the severe acute respiratory syndrome. Jama. 2003; 290: 374–80. 10.1001/jama.290.3.374 12865379

[64] Almekhlafi GA, Albarrak MM, Mandurah Y, et al Presentation and outcome of the Middle East respiratory syndrome in Saudi intensive care unit patients. Critical Care. 2016; 20: 123 10.1186/s13054-016-1303-8 27153800 PMC4859954

[65] Garbati MA, Fagbo SF, Fang VJ, et al A comparative study of clinical presentation and risk factors for adverse outcomes in patients hospitalized with acute respiratory disease due to MERS coronavirus or other causes. PloS one. 2016; 11. 10.1371/journal.pone.0165978 PMC5094725 27812197

[66] Al Ghamdi M, Alghamdi KM, Ghandoora Y, et al Treatment outcomes for patients with Middle Eastern Respiratory Syndrome Coronavirus (MERS CoV) infection at a coronavirus referral center in the Kingdom of Saudi Arabia. BMC infectious diseases. 2016; 16: 174 10.1186/s12879-016-1492-4 27097824 PMC4839124

[67] Aleanizy FS, Mohmed N, Alqahtani FY, et al Outbreak of Middle East respiratory syndrome coronavirus in Saudi Arabia: a retrospective study. BMC infectious diseases. 2017; 17: 23 10.1186/s12879-016-2137-3 28056850 PMC5217314

[68] Aya AG, Vialles N, Tanoubi I, et al Spinal anesthesia-induced hypotension: a risk comparison between patients with severe preeclampsia and healthy women undergoing preterm cesarean delivery. Anesthesia & Analgesia. 2005; 101: 869–75. 16116006 10.1213/01.ANE.0000175229.98493.2B

