# Supplementary material for: Rate of Intensive Care Unit admission and outcomes among patients with coronavirus: A systematic review and Meta-analysis
Source: PLoS One. 2020 Jul 10;15(7):e0235653. doi: 10.1371/journal.pone.0235653 (PMC7351172; doi:10.1371/journal.pone.0235653)
Supplement: S1 Table — (DOCX) [file pone.0235653.s001.docx]

### Supplemental Table 1: description of excluded studies with reasons

| Author | Year of publication | Country | Reason for exclusion |
| --- | --- | --- | --- |
| Hasan et al | 2016 | Saudi Arabia | It is a clinical review |
| Jiang et al | 2019 | China | Study on psychosocial impacts of COVID-19 |
| Wang et al | 2006 | China | Review on bats and SARS |
| Chen et al | 2020 | China | Different outcome variables |
| Alserehi et al | 2016 | Saudi Arabia | Case report |
| Lau et al | 2015 | China | Different outcomes of interest |
| Aleanizy et al | 2017 | Saudi Arabia | Outcome of interest not reported |
| Fan et al | 2020 | China | Different Outcome of interest |
| Al-Dorzi et al | 2026 | Saudi Arabia | Clinical review |
| Vandroux et al | 2018 | France | Short communication on Coronavirus OC43 |
| Lai et al | 2019 | China | Clinical review |
| Manocha et al | 2003 | Canada | Review |
| McMichael et al | 2020 | USA | Different outcome of interest |
