# Supplementary material for: Rate of Intensive Care Unit admission and outcomes among patients with coronavirus: A systematic review and Meta-analysis
Source: PLoS One. 2020 Jul 10;15(7):e0235653. doi: 10.1371/journal.pone.0235653 (PMC7351172; doi:10.1371/journal.pone.0235653)
Supplement: S2 Table — (DOCX) [file pone.0235653.s002.docx]

## **Supplemental table 2 methodological quality of included studies**

| Author(s) | Q1 | Q2 | Q3 | Q4 | Q5 | Q6 | Q7 | Q8 | Score |
| --- | --- | --- | --- | --- | --- | --- | --- | --- | --- |
| Liu et al([1](#_ENREF_1)) | Y | Y | Y | Y | Y | Y | Y | Y | 8 |
| Xu et al([2](#_ENREF_2)) | Y | Y | Y | Y | Y | N | Y | N | 6 |
| Arentz et al([3](#_ENREF_3)) | Y | Y | Y | Y | N | N | Y | N | 5 |
| Bhatraju et al([4](#_ENREF_4)) | Y | N | Y | Y | N | N | Y | Y | 5 |
| Bialek et al([5](#_ENREF_5)) | Y | Y | Y | Y | N | N | Y | N | 5 |
| Cao et al([6](#_ENREF_6)) | Y | Y | Y | Y | N | N | N | N | 4 |
| Chen et al([7](#_ENREF_7)) | Y | Y | Y | Y | Y | N | Y | N | 6 |
| Chen et al([8](#_ENREF_8)) | Y | Y | Y | Y | Y | Y | Y | Y | 8 |
| Huang et al([9](#_ENREF_9)) | Y | Y | Y | Y | Y | N | Y | N | 6 |
| Petrilli et al([10](#_ENREF_10)) | N | N | Y | Y | Y | Y | Y | Y | 6 |
| Richardson et al([11](#_ENREF_11)) | N | Y | Y | Y | Y | Y | Y | Y | 7 |
| Simonnet et al([12](#_ENREF_12)) | N | Y | Y | N | Y | Y | N | Y | 5 |
| Wang et al([13](#_ENREF_13)) | Y | Y | Y | Y | N | Y | N | Y | 6 |
| Wu et al([14](#_ENREF_14)) | N | N | Y | Y | Y | Y | Y | Y | 6 |
| Yang et al([15](#_ENREF_15)) | Y | Y | Y | Y | Y | N | Y | N | 6 |
| Young et al([16](#_ENREF_16)) | Y | Y | Y | Y | Y | N | Y | N | 6 |
| Guan et al([17](#_ENREF_17)) | N | Y | Y | Y | N | Y | Y | Y | 6 |
| Zhou et al([18](#_ENREF_18)) | N | Y | N | Y | Y | Y | Y | y | 6 |
| Lodigiania et al([19](#_ENREF_19)) | Y | Y | N | Y | Y | Y | Y | Y | 7 |
| Kloka et al([20](#_ENREF_20)) | N | Y | Y | N | N | Y | Y | Y | 5 |
| Lei et al ([21](#_ENREF_21)) | N | Y | Y | Y | Y | N | Y | Y | 6 |
| Docherty et al([22](#_ENREF_22)) | N | Y | Y | Y | N | Y | Y | Y | 6 |
| Du et al ([23](#_ENREF_23)) | N | Y | Y | Y | N | N | Y | Y | 5 |
| Ling et al([24](#_ENREF_24)) | N | Y | Y | N | N | N | Y | Y | 5 |
| Zangrillo et al ([25](#_ENREF_25)) | N | Y | Y | N | N | N | Y | Y | 4 |
| Grasselli et al ([26](#_ENREF_26)) | N | Y | Y | Y | Y | N | Y | Y | 6 |
| Chan et al([27](#_ENREF_27)) | Y | Y | Y | Y | Y | N | Y | Y | 7 |
| Chen et al([28](#_ENREF_28)) | N | Y | Y | Y | N | N | Y | Y | 5 |
| Choi et al([29](#_ENREF_29)) | Y | Y | Y | Y | Y | Y | Y | Y | 8 |
| Lew TW et al([30](#_ENREF_30)) | Y | Y | Y | Y | Y | Y | Y | Y | 8 |
| Almekhlafie GA et al([31](#_ENREF_31)) | Y | Y | Y | Y | N | Y | N | Y | 6 |
| Al-Hameed et al([32](#_ENREF_32)) | Y | Y | Y | Y | N | Y | N | Y | 6 |
| Garbati et al([33](#_ENREF_33)) | Y | Y | Y | Y | Y | Y | Y | Y | 8 |
| Al Ghamdi et al([34](#_ENREF_34)) | Y | Y | Y | Y | N | N | Y | Y | 5 |
| Halim et al([35](#_ENREF_35)) | Y | Y | Y | Y | Y | N | Y | Y | 7 |
| Saad et al([36](#_ENREF_36)) | Y | Y | Y | Y | Y | Y | Y | Y | 8 |
| Arabi YM et al([37](#_ENREF_37)) | Y | Y | Y | Y | N | N | Y | Y | 6 |

Q: question; Y: yes; N: No

1. Liu W, Tao Z-W, Wang L, Yuan M-L, Liu K, Zhou L, et al. Analysis of factors associated with disease outcomes in hospitalized patients with 2019 novel coronavirus disease. Chinese medical journal. 2020.

2. Xu X-W, Wu X-X, Jiang X-G, Xu K-J, Ying L-J, Ma C-L, et al. Clinical findings in a group of patients infected with the 2019 novel coronavirus (SARS-Cov-2) outside of Wuhan, China: retrospective case series. bmj. 2020;368.

3. Arentz M, Yim E, Klaff L, Lokhandwala S, Riedo FX, Chong M, et al. Characteristics and outcomes of 21 critically ill patients with COVID-19 in Washington State. Jama. 2020;323(16):1612-4.

4. Bhatraju PK, Ghassemieh BJ, Nichols M, Kim R, Jerome KR, Nalla AK, et al. Covid-19 in critically ill patients in the Seattle region—case series. New England Journal of Medicine. 2020;382(21):2012-22.

5. COVID C, Team R. Severe outcomes among patients with coronavirus disease 2019 (COVID-19)—United States, February 12–March 16, 2020. MMWR Morb Mortal Wkly Rep. 2020;69(12):343-6.

6. Cao J, Hu X, Cheng W, Yu L, Tu W-J, Liu Q. Clinical features and short-term outcomes of 18 patients with corona virus disease 2019 in intensive care unit. Intensive care medicine. 2020:1-3.

7. Chen J, Qi T, Liu L, Ling Y, Qian Z, Li T, et al. Clinical progression of patients with COVID-19 in Shanghai, China. Journal of Infection. 2020.

8. Chen C-Y, Lee C-H, Liu C-Y, Wang J-H, Wang L-M, Perng R-P. Clinical features and outcomes of severe acute respiratory syndrome and predictive factors for acute respiratory distress syndrome. Journal of the Chinese Medical Association. 2005;68(1):4-10.

9. Huang C, Wang Y, Li X, Ren L, Zhao J, Hu Y, et al. Clinical features of patients infected with 2019 novel coronavirus in Wuhan, China. The Lancet. 2020;395(10223):497-506.

10. Petrilli CM, Jones SA, Yang J, Rajagopalan H, O'Donnell LF, Chernyak Y, et al. Factors associated with hospitalization and critical illness among 4,103 patients with COVID-19 disease in New York City. MedRxiv. 2020.

11. Richardson S, Hirsch JS, Narasimhan M, Crawford JM, McGinn T, Davidson KW, et al. Presenting characteristics, comorbidities, and outcomes among 5700 patients hospitalized with COVID-19 in the New York City area. Jama. 2020.

12. Simonnet A, Chetboun M, Poissy J, Raverdy V, Noulette J, Duhamel A, et al. High prevalence of obesity in severe acute respiratory syndrome coronavirus‐2 (SARS‐CoV‐2) requiring invasive mechanical ventilation. Obesity. 2020.

13. Wang D, Hu B, Hu C, Zhu F, Liu X, Zhang J, et al. Clinical characteristics of 138 hospitalized patients with 2019 novel coronavirus–infected pneumonia in Wuhan, China. Jama. 2020;323(11):1061-9.

14. Wu C, Chen X, Cai Y, Zhou X, Xu S, Huang H, et al. Risk factors associated with acute respiratory distress syndrome and death in patients with coronavirus disease 2019 pneumonia in Wuhan, China. JAMA internal medicine. 2020.

15. Yang X, Yu Y, Xu J, Shu H, Liu H, Wu Y, et al. Clinical course and outcomes of critically ill patients with SARS-CoV-2 pneumonia in Wuhan, China: a single-centered, retrospective, observational study. The Lancet Respiratory Medicine. 2020.

16. Young BE, Ong SWX, Kalimuddin S, Low JG, Tan SY, Loh J, et al. Epidemiologic features and clinical course of patients infected with SARS-CoV-2 in Singapore. Jama. 2020;323(15):1488-94.

17. Guan W-j, Liang W-h, Zhao Y, Liang H-r, Chen Z-s, Li Y-m, et al. Comorbidity and its impact on 1590 patients with Covid-19 in China: A Nationwide Analysis. European Respiratory Journal. 2020;55(5).

18. Zhou F, Yu T, Du R, Fan G, Liu Y, Liu Z, et al. Clinical course and risk factors for mortality of adult inpatients with COVID-19 in Wuhan, China: a retrospective cohort study. The Lancet. 2020.

19. Lodigiani C, Iapichino G, Carenzo L, Cecconi M, Ferrazzi P, Sebastian T, et al. Venous and arterial thromboembolic complications in COVID-19 patients admitted to an academic hospital in Milan, Italy. Thrombosis research. 2020.

20. Klok FA, Kruip M, Van Der Meer N, Arbous M, Gommers D, Kant K, et al. Confirmation of the high cumulative incidence of thrombotic complications in critically ill ICU patients with COVID-19: an updated analysis. Thrombosis research. 2020.

21. Lei S, Jiang F, Su W, Chen C, Chen J, Mei W, et al. Clinical characteristics and outcomes of patients undergoing surgeries during the incubation period of COVID-19 infection. EClinicalMedicine. 2020:100331.

22. Docherty AB, Harrison EM, Green CA, Hardwick HE, Pius R, Norman L, et al. Features of 20 133 UK patients in hospital with covid-19 using the ISARIC WHO Clinical Characterisation Protocol: prospective observational cohort study. bmj. 2020;369.

23. Du R-H, Liu L-M, Yin W, Wang W, Guan L-L, Yuan M-L, et al. Hospitalization and critical care of 109 decedents with COVID-19 pneumonia in Wuhan, China. Annals of the American Thoracic Society. 2020(ja).

24. Ling L, So C, Shum HP, Chan PK, Lai CK, Kandamby DH, et al. Critically ill patients with COVID-19 in Hong Kong: a multicentre retrospective observational cohort study. Crit Care Resusc. 2020;6.

25. Zangrillo A, Beretta L, Scandroglio AM, Monti G, Fominskiy E, Colombo S, et al. Characteristics, treatment, outcomes and cause of death of invasively ventilated patients with COVID-19 ARDS in Milan, Italy. Crit Care Resusc. 2020.

26. Grasselli G, Zangrillo A, Zanella A, Antonelli M, Cabrini L, Castelli A, et al. Baseline characteristics and outcomes of 1591 patients infected with SARS-CoV-2 admitted to ICUs of the Lombardy Region, Italy. Jama. 2020;323(16):1574-81.

27. Chan J, Ng C, Chan Y, Mok T, Lee S, Chu S, et al. Short term outcome and risk factors for adverse clinical outcomes in adults with severe acute respiratory syndrome (SARS). Thorax. 2003;58(8):686-9.

28. Chen N, Zhou M, Dong X, Qu J, Gong F, Han Y, et al. Epidemiological and clinical characteristics of 99 cases of 2019 novel coronavirus pneumonia in Wuhan, China: a descriptive study. The Lancet. 2020;395(10223):507-13.

29. Choi KW, Chau TN, Tsang O, Tso E, Chiu MC, Tong WL, et al. Outcomes and prognostic factors in 267 patients with severe acute respiratory syndrome in Hong Kong. Annals of internal medicine. 2003;139(9):715-23.

30. Lew TW, Kwek T-K, Tai D, Earnest A, Loo S, Singh K, et al. Acute respiratory distress syndrome in critically ill patients with severe acute respiratory syndrome. Jama. 2003;290(3):374-80.

31. Almekhlafi GA, Albarrak MM, Mandourah Y, Hassan S, Alwan A, Abudayah A, et al. Presentation and outcome of Middle East respiratory syndrome in Saudi intensive care unit patients. Critical Care. 2016;20(1):123.

32. Al-Hameed F, Wahla AS, Siddiqui S, Ghabashi A, Al-Shomrani M, Al-Thaqafi A, et al. Characteristics and outcomes of Middle East respiratory syndrome coronavirus patients admitted to an intensive care unit in Jeddah, Saudi Arabia. Journal of intensive care medicine. 2016;31(5):344-8.

33. Garbati MA, Fagbo SF, Fang VJ, Skakni L, Joseph M, Wani TA, et al. A comparative study of clinical presentation and risk factors for adverse outcome in patients hospitalised with acute respiratory disease due to MERS coronavirus or other causes. PloS one. 2016;11(11).

34. Al Ghamdi M, Alghamdi KM, Ghandoora Y, Alzahrani A, Salah F, Alsulami A, et al. Treatment outcomes for patients with Middle Eastern Respiratory Syndrome Coronavirus (MERS CoV) infection at a coronavirus referral center in the Kingdom of Saudi Arabia. BMC infectious diseases. 2016;16(1):174.

35. Halim AA, Alsayed B, Embarak S, Yaseen T, Dabbous S. Clinical characteristics and outcome of ICU admitted MERS corona virus infected patients. Egyptian Journal of Chest Diseases and Tuberculosis. 2016;65(1):81-7.

36. Saad M, Omrani AS, Baig K, Bahloul A, Elzein F, Matin MA, et al. Clinical aspects and outcomes of 70 patients with Middle East respiratory syndrome coronavirus infection: a single-center experience in Saudi Arabia. International Journal of Infectious Diseases. 2014;29:301-6.

37. Arabi YM, Arifi AA, Balkhy HH, Najm H, Aldawood AS, Ghabashi A, et al. Clinical course and outcomes of critically ill patients with Middle East respiratory syndrome coronavirus infection. Annals of internal medicine. 2014;160(6):389-97.
