# Supplementary material for: Rate of Intensive Care Unit admission and outcomes among patients with coronavirus: A systematic review and Meta-analysis
Source: PLoS One. 2020 Jul 10;15(7):e0235653. doi: 10.1371/journal.pone.0235653 (PMC7351172; doi:10.1371/journal.pone.0235653)
Supplement: S2 Fig — ICU: Intensive Care Unit. (DOCX) [file pone.0235653.s004.docx]

**Supplemental Fig2:** Forest plot for subgroup analysis of prevalence of ICU comorbidity by types of coronavirus: The midpoint of each line illustrates the prevalence; the horizontal line indicates the confidence interval, and the diamond shows the pooled prevalence. ICU: Intensive Care Unit
