# Supplementary material for: Rate of Intensive Care Unit admission and outcomes among patients with coronavirus: A systematic review and Meta-analysis
Source: PLoS One. 2020 Jul 10;15(7):e0235653. doi: 10.1371/journal.pone.0235653 (PMC7351172; doi:10.1371/journal.pone.0235653)
Supplement: S4 Fig — (DOCX) [file pone.0235653.s006.docx]

**Supplemental Fig 4:** Forest plot showing pooled odds ratio (log scale) of the associations between Intensive Care Unit mortality and its determinants (A: Co-morbidities; B: Age greater than 50 years; C: Gender D: ARDS).
