# Supplementary material for: Rate of Intensive Care Unit admission and outcomes among patients with coronavirus: A systematic review and Meta-analysis
Source: PLoS One. 2020 Jul 10;15(7):e0235653. doi: 10.1371/journal.pone.0235653 (PMC7351172; doi:10.1371/journal.pone.0235653)
Supplement: S1 Checklist — (DOC) [file pone.0235653.s007.doc]

| **Section/topic** | **#** | **Checklist item** | **Reported on page #** |
| --- | --- | --- | --- |
| **TITLE** | | |  |
| Title | 1 | Rate of Intensive Care Unit admission and outcomes among patients with coronavirus: A systematic review and Meta-analysis | 1 |
| **ABSTRACT** | | |  |
| Structured summary | 2 | Abstract **Background:** The rate of ICU admission among patients with coronavirus varied from 3% to 100% and the mortality was as high as 86% of admitted patients. The objective of the systematic review was to investigate the rate of ICU admission, mortality, morbidity and complications among patients with coronavirus.  **Methods:** A comprehensive strategy was conducted in PubMed/Medline; Science direct and LILACS from December, 2002 to May, 2020 without language restriction. The Heterogeneity among the included studies was checked with forest plot, χ2 test, I2 test, and the p-values. All observational studies reporting rate of ICU admission, prevalence of mortality and its determinants among ICU admitted patients with coronavirus were included and the rest were excluded  **Result:** A total of 646 articles were identified from different databases and 50 articles were selected for evaluation. Thirty-seven Articles with 24983 participants were included. The rate of ICU admission was 32% (95% CI: 26 to 38, 37 studies and 32, 741 participants). The Meta-Analysis revealed that the pooled prevalence of mortality in patients with coronavirus disease in ICU was 39% (95% CI: 34 to 43, 37 studies and 24, 983 participants).  **Conclusion:** The Meta-Analysis revealed that approximately one-third of patients admitted to ICU with severe Coronavirus disease and more than thirty percent of patients admitted in ICU with a severe form of COVID-19 for better care died which warns the health care stakeholders to give attention to intensive care patients.  **Registration:** This Systematic review and Meta-Analysis was registered in Prospero international prospective register of systemic reviews (CRD42020177095) on April 9/2020.  **Keywords:** Coronavirus, COVID-19, severe acute respiratory syndrome, mortality | 2 |
| **INTRODUCTION** | | |  |
| Rationale | 3 | The global rates of Intensive Care Unit admission, number of cases requiring ICU care, number of cases need a mechanical ventilator, the prevalence of death in ICU, length of stay and independent risk factors for in-hospital mortality are very important variables to be determined to reduce patient mortality and morbidity through varies strategies including but not limited to increasing number of ICU beds, mechanical ventilator, skilled professionals, integrated monitors and reducing possible risk factors. | 4 |
| Objectives | 4 | This systematic review aimed to provide global evidence on rates of ICU admission and outcomes among patients with coronavirus. | 4 |
| **METHODS** | | |  |
| Protocol and registration | 5 | This Systematic Review and Meta-Analysis was registered in Prospero international prospective register of systemic reviews (CRD42020177095) on April 9/2020. | 5 |
| Eligibility criteria | 6 | All cross-sectional studies reporting rate of ICU admission and ICU mortality will be included and the rest were excluded | 5 |
| Information sources | 7 | The search strategy was intended to explore all available published and unpublished studies among Coronaviruses infected patients admitted to ICU from December 2002 to May2020 language restrictions. A three steps search strategy was employed in this review. An initial search on PubMed/Medline, Science direct and African Online Journal was carried out followed by an analysis of the text words contained in Title/Abstract and indexed terms. A second search was undertaken by combining free text words and indexed terms with Boolean operators. The third search was conducted with the reference lists of all identified reports and articles for additional studies. Finally, an additional and grey literature search was conducted on Google scholars. | 5 |
| Search | 8 | PubMed/Medline, Science Direct and Cochrane library were searched for all possible Mesh terms and PICOs as SARS[Title/Abstract]) OR (SARS-COV-2[Title/Abstract])) OR (COVID-19[Title/Abstract])) AND (MERS[Title/Abstract])) AND (mortality[Title/Abstract])) OR (morbidity[Title/Abstract])) AND (ICU[Title/Abstract])) OR (hospital[Title/Abstract])) AND (prevalence[Title/Abstract])) AND (risk factors[Title/Abstract])). | 6 |
| Study selection | 9 | Observational studies reporting rates of ICU admission and /or mortality in patients with coronavirus from December 2002 to MAY 30, 2020, without language restriction, were selected | 6 |
| Data collection process | 10 | Data were extracted independently with two authors with customized format excel sheet and any disagreement was resolved by the other authors | 6 |
| Data items | 11 | The extracted data includes: author, year of publication, study period, event, sample size, country, risk factors of mortality for factor analysis | 6 |
| Risk of bias in individual studies | 12 | The methodological quality of each study was evaluated with tools adopted from Joanna Briggs institute for Mata-analysis of cross-sectional studies | 6 |
| Summary measures | 13 | The primary summary measure was prevalence | 6 |
| Synthesis of results | 14 | The meta-analysis was done with a random effect model as there was significant heterogeneity as depicted with I2  Furthermore, subgroup analysis was done to identify the source of heterogeneity | 6 |

Page 1 of 2

| **Section/topic** | **#** | **Checklist item** | **Reported on page #** |
| --- | --- | --- | --- |
| Risk of bias across studies | 15 | Funnel plot didn’t show publication bias and rank correlation and eggers linear regression didn’t show significant publication bias | 6 |
| Additional analyses | 16 | Subgroup analysis, sensitivity analysis, and moderator analysis were done | 6 |
| **RESULTS** | | |  |
| Study selection | 17 | This part was displayed with a flow diagram in the result part | 8 |
| Study characteristics | 18 | Description of included studies were described with table | 9 |
| Risk of bias within studies | 19 | Methodological quality assessment was done | 10 |
| Results of individual studies | 20 | Results of included studies were described in the result section | 10 |
| Synthesis of results | 21 | Twenty-two studies reporting rate of ICU admission and outcomes were included | 11 |
| Risk of bias across studies | 22 | Funnel plot along with eager and begs test | 18 |
| Additional analysis | 23 | Subgroup analysis, sensitivity analysis, and factor analysis | 18 |
| **DISCUSSION** | | |  |
| Summary of evidence | 24 | The rate of ICU admission was 32% (95% CI: 26 to 38, 37 studies and 32, 741 participants). The Meta-Analysis revealed that the pooled prevalence of mortality in patients with coronavirus disease in ICU was 39% (95% CI: 34 to 43, 37 studies and 24, 983 participants). | 19 |
| Limitations | 25 | The review incorporated plenty of studies with a large number of participants but the majority of studies included in this review didn’t report data on comorbidity and risk factors to investigate the independent predictors. Besides, there were a limited number of studies in some countries and it would be difficult to provide conclusive evidence with results pooled from fewer studies. | 20 |
| Conclusions | 26 | The systematic review and Meta-Analysis revealed that approximately one-third of patients admitted to ICU with severe Coronavirus disease. The systematic review also showed that more than thirty percent of patients admitted in ICU with a severe form of COVID-19 for better care died which warns the health care stakeholders to give attention to intensive care patients admitted with COVID-19 through accessing mechanical ventilators, integrated patient monitors, skilled ICU staffs, creation of awareness about infection prevention and more others. Besides, prevalence of mortality had strong relation with comorbidity, age, gender, and complication. | 21 |
| **FUNDING** | | |  |
| Funding | 27 | Authors own resources | 22 |
