# Supplementary material for: Rate of Intensive Care Unit admission and outcomes among patients with coronavirus: A systematic review and Meta-analysis
Source: PLoS One. 2020 Jul 10;15(7):e0235653. doi: 10.1371/journal.pone.0235653 (PMC7351172; doi:10.1371/journal.pone.0235653)
Supplement: Attachment — Submitted filename: response to reviewer comments.docx [file pone.0235653.s008.docx]

| Section | Correction | Page # | Lines |
| --- | --- | --- | --- |
| Title | Rate of Intensive Care Unit admission and outcomes among patients with coronavirus: A systematic review and Meta-analysis | 1 |  |
| Abstract | Corrected as per the reviewers suggestions and changes were highlighted | 2 | 25-43 |
| Background | Corrected as per reviewer comment, but description of types of coronavirus along with some epidemiological aspect is very important for a reader that was why those data were incorporated. | 3 | 61-82 |
| Background | The previous search was conducted early in April and the data on COVID-19 were outdated. Recent background information is incorporated. The pandemic is increasing in Africa rapidly but there is no studies published so far except WHO and health ministry’s reports | 3 | 61-85 |
| Background | The objective was stated at the last paragraph of the background. | 4 | 107-110 |
| Methods | Very important comments to be addressed to meet the journal guidelines and standards. All the concerns are tried to address. Additional search was conducted to incorporate recently published articles. Hence, 15 additional studies were retrieved. | 5-6 | 118-71 |
| Results | Corrected as per reviewers’ suggestions | 7-9 | 3178-239 |
| Results | Meta-analysis was done on rate of ICU admission, prevalence of mortality, comorbidity and complication. Subgroup analysis was done by country, types of coronavirus, type comorbidity and complication. Factor analysis was conducted with age, gender, comorbidity and more prevalent complication (ARDS). influential analysis and publication bias was done as well in R with metafor package and the comments were addressed | 11-18 | 245-344 |
| Discussion | Corrected as per the reviewer suggestions. Overall quality of evidence, limitation implication for practice research were included as well | 19-20 | 345-402 |
| Conclusion | Corrected as per the reviewer comment | 21 | 405-11 |
| Declaration | Editorial comments were addressed. | 21 | ------ |
| References | References are added and removed to suit the current revised document |  |  |
